# Associations of SNPs located at candidate genes to bovine growth traits, prioritized with an interaction networks construction approach

**DOI:** 10.1186/s12863-015-0247-3

**Published:** 2015-07-22

**Authors:** Francisco Alejandro Paredes-Sánchez, Ana María Sifuentes-Rincón, Aldo Segura Cabrera, Carlos Armando García Pérez, Gaspar Manuel Parra Bracamonte, Pascuala Ambriz Morales

**Affiliations:** Laboratorio de Biotecnología Animal, Centro de Biotecnología Genómica. IPN, Boulevard del Maestro esq. Elías Piña, Col. Narciso Mendoza, Cd. Reynosa, Tam C.P. 88710 Mexico; Laboratorio de Bioinformática, Centro de Biotecnología Genómica. IPN, Boulevard del Maestro esq. Elías Piña, Col. Narciso Mendoza, Cd. Reynosa, Tam C.P. 88710 Mexico; Red de Estudios Moleculares Avanzados, Instituto de Ecología, A.C., Xalapa, Mexico

**Keywords:** Candidate gene approach, Genetic networks, Beef cattle, Economically relevant traits

## Abstract

**Background:**

For most domestic animal species, including bovines, it is difficult to identify causative genetic variants involved in economically relevant traits. The candidate gene approach is efficient because it investigates genes that are expected to be associated with the expression of a trait and defines whether the genetic variation present in a population is associated with phenotypic diversity. A potential limitation of this approach is the identification of candidates. This study used a bioinformatics approach to identify candidate genes via a search guided by a functional interaction network.

**Results:**

A functional interaction network tool, BosNet, was constructed for *Bos taurus*. Predictions for candidate genes were performed using the guilt-by-association principle in BosNet. Association analyses identified five novel markers within BosNet-prioritized genes that had significant effects on different growth traits in Charolais and Brahman cattle.

**Conclusions:**

BosNet is an excellent tool for the identification of single nucleotide polymorphisms that are potentially associated with complex traits.

## Background

In bovines, most economically relevant traits (ERTs) are considered to be genetically complex traits; therefore, different approaches have been utilized to identify genetic variation related to phenotypic differences. However, identifying causative genetic variants involved in ERT phenotypes is a difficult task.

Although the genome-wide association approach has become the most frequently applied strategy to identify genetic variation that explains ERTs, the candidate gene approach has also been widely used to identify genetic variation. The candidate gene strategy is efficient because it investigates genes that are expected to be associated with the expression of a trait and defines whether the genetic variation present in populations is associated with phenotypic diversity [1]. In an association study, two of the critical steps used in the candidate gene approach are selecting a suitable candidate gene and identifying the most useful genetic variants or polymorphisms (if known) for testing.

Traditionally, physiological function, positional cloning and comparative genomic approaches have been used to select candidate genes [2–6]; however, interaction network analysis may also be an excellent alternative to selecting candidate genes for ERTs in bovine. Lim et al. [7] constructed a protein-protein interaction (PPI) network to identify candidate genes for marbling traits in bovines. These authors successfully identified candidate genes associated with intramuscular fat and suggested that the PPI approach can be used to identify biological pathways and regulatory elements involved in marbling-related genes.

The guilt-by-association strategy uses biological information available in databases and statistical methods to identify potential candidate genes *in silico*. This approach searches for candidate genes based on their interactions with a set of reference genes (genes previously associated with a phenotype) [8]. This approach is based on the tendency of genes associated with the same biological process to interact within a network and organize themselves in modules or functional groups. Within these modules, new candidate genes can be identified, and gene interactions can be analyzed with a set of reference genes (genes previously associated with a phenotype). Based on these interactions, it is likely that these genes will be strongly associated with the set of reference genes and that the single nucleotide polymorphisms (SNPs) in which they are found will be involved in the same biological processes.

Hence, animal science has begun to utilize bioinformatics to model and generate interaction networks that represent the architectural genetics of complex traits in bovines, such as marbling, age at puberty and reproductive characteristics [7, 9, 10]. The objectives of this work were to develop BosNet as a tool for the identification and prioritization of genes associated with complex traits and to assess the efficiency of the BosNet tool in associating SNPs located on BosNet-prioritized genes with bovine growth traits.

## Results

### Modeled networks for B. taurus

A highly reliable integrated network was constructed for *Bos taurus.* By identifying orthologous genes, 16,348 new annotations were obtained for bovine genes that were previously lacking annotations, and their combination with known annotations (34,082) resulted in 50,380 annotations for *B. taurus* genes. The increased number of functional annotations was used to obtain an integrated network referred to as BosNet. This network consists of 1,747,160 associations among 16,065 genes, which is equivalent to 73 % coverage of the bovine genome. BosNet can be freely consulted at http://www.cbg.ipn.mx/investigacion/Paginas/BosNet.aspx. In the current version of BosNet (March 2015), the number of Gene Ontology annotations in the BP (Biological Process) domain has increased by 113 % over the 2012 version of BosNet. The current version consists of 4.19825 million interactions and has 20 % greater *B. taurus* genome coverage.

By using a text mining approach, 60 genes associated with different parameters related to bovine growth traits were identified. This information permitted an immediate evaluation of the individual contribution of each of the networks for *B. taurus* to correctly identify genes previously associated with bovine growth. This ability was characterized by receiver operating characteristic (ROC) curves. The area under the curve (AUC) was used as an indicator of the predictive power of each network. The performance of each network modeled from different databases was reduced compared with the performance obtained from the integrated network, indicating that the use of these networks independently reduces both the predictive power and coverage.

### Identification and prioritization of candidate genes for growth traits and gene variability in bovine breeds

In the analysis conducted using the BosNet network, the positive predictive value (PPV) was calculated by establishing that all of the genes with an associated score ≥ 39.6468 had a 53 % probability of being associated with the growth trait. The genes that met this condition included *RXRA* (retinoid X receptor alpha), *IGF1R* (insulin-like growth factor 1 receptor), *TCF15* (transcription factor 15), *INS* (insulin), *USF1* (upstream transcription factor 1) and *EGFR* (epidermal growth factor receptor).

These genes were used as targets to determine variations in SNPs, which were used in association studies of bovine growth traits. Three new *INS* gene polymorphisms were identified (g.50,036,892 G > A; C > T g.50,036,987 and g.50,037,033 A > G). Five *USF1* gene SNPs were identified with four transitions and one indel (insertion-deletion polymorphism). The g.8,458,558 A > G, g.8,458,837 G > A, g.8,459,971 A > G, g.8,460,354 C > T and g.8,460,878 C > T SNPs are located in intron 2, intron 3, intron 6, exon 8 and intron 9, respectively. The g.8,459,028 -/C indel is located in intron 3. For the *TCF15* gene, the analysis only revealed the presence of one SNP (g.60,997,442 G > A), which corresponds to a transition located within intron 1. The *RXRA* gene demonstrated the highest SNP variation, with a total of 34 SNPs distributed throughout the gene. Of these SNPs, 25 are located in introns, including six transversions. The remaining eight SNPs are located in coding regions, and the most significant is a transversion located in exon 3.

Novel SNPs and GenBank-reported SNPs in the coding regions of the six genes were used for genotyping in two bovine populations. Of the tested SNPs, 70 % and 50 % were monomorphic in the Charolais and Brahman populations, respectively. The allelic frequencies from the polymorphic SNPs are presented in Table 1.

**Table 1:** Allele frequencies of SNPs located in BosNet-prioritized genes

Breed	Gene	SNP_ID	A	C	G	T
Charolais	EGFR	rs11004527		0.5217		0.4783
		rs13687792		0.4275		0.5725
		rs21017031		0.1413		0.8587
		rs21165825		0.9312		0.0688
		rs37921750	0.2283		0.7717	
		rs38513127	0.2681		0.7319	
	IGF1R	rs13486888		0.5657		0.4343
		rs20814099		0.6957		0.3043
		rs21077860		0.5438		0.4562
		rs38090000	0.0725		0.9275	
		rs4164070	0.1739		0.8261	
		rs4196133		0.8514		0.1486
	INS	rs10949071		0.7799		0.2201
	RXRA	g.105,985,027			0.5833	0.4167
		g.105,985,044			0.3496	0.6504
		g.106,004,449		0.3664		0.6336
		g.105,986,715			0.9565	0.0435
	TCF15	g.60,997,442	0.3043			0.6957
Brahman	EGFR	rs11004527		0.1187		0.8812
		rs13687792		0.1415		0.8585
		rs21017031		0.8738		0.1262
		rs37921750	0.0613		0.9387	
		rs38513127	0.0619		0.9381	
	IGF1R	rs13486888		0.9159		0.0841
		rs20814099		0.8679		0.1321
		rs20973667		0.1682		0.8318
		rs378266791		0.5685		0.4315
	INS	g.50,037,033	0.0896		0.9104	
		g.50,036,892	0.2404		0.7596	
		g.50,036,987		0.3396		0.6604
	RXRA	g.106,004,142	0.5054		0.4946	
		g.106,004,147		0.5841		0.4159
		g.105,986,149	0.425		0.575	
		g.105,989,179	0.4074		0.568	
		g.106,004,180	0.7938		0.2062	
		g.106,004,184	0.3586		0.6414	
		g.105,989,219		0.8048		0.1952
		g.105,989,022		0.4112		0.5888
		g.105,990,023	0.1898	0.8102		
		g.105,989,236		0.8102		0.1898
		g.106,011,253		0.4348		0.5652
		g.105,985,027			0.0521	0.9479
		g.105,985,044			0.7128	0.2872
		g.106,004,449		0.6311		0.3689
		g.106,004,518	0.5728	0.4272		
		g.106,011,539	0.6038		0.3962	
		g. 105,990,568	0.82		0.18	
		g.105,989,080		0.4151		0.5849
		rs13628911		0.875		0.125
	TCF15	g.60,997,442	0.0888		0.9112	
	USF1	g.8,458,558	0.2286		0.7714	
		g.8,458,837	0.774		0.226	
		g.8,459,971	0.2667		0.7333	
		g.8,460,354		0.226		0.774

*BW* Birth weight, *WW* Weaning weight, *YW* Yearling weight, *FS* Frame size. *MC* mean comparison, means with different letter are significantly different (P < 0.05). *P < 0.05; **P < 0.01

### Association of novel SNPs with growth traits in Charolais and Brahman cattle

We tested the ability of the BosNet tool to prioritize candidate genes by detecting associations between quantitative trait loci and growth traits in Charolais and Brahman cattle.

In the Brahman population, the association analysis demonstrated that only rs136289117 located in the *RXRA* gene had a significant effect (p *=* 0.0394) on weaning weight (WW). The heterozygous genotype mean WW (215.029 kg) was approximately 10 kg higher than that of the homozygous CC genotype (206.152 kg).

For Charolais cattle, the association analysis resulted in four novel SNPs that were significantly associated with growth traits (P ≤ 0.04) (Table 2). The TT genotype of the rs210778604 SNP in the *IGF1* receptor gene had a significant effect on birth weight (BW), which was 2.5 kg higher than the BW of the heterozygous (CT) and homozygous (CC) genotypes (Table 2). Interestingly, this same locus was significantly related to frame size (FS). The favorable CC genotype produced slightly taller animals (P = 0.0195). The g.106,0040,449 marker located in the *RXRA* gene was significantly associated with WW. The WW of animals with the CT genotype was approximately 21 kg higher than that of homozygous TT animals (P *=* 0.0028). The same marker was associated with yearling weight (YW); animals with the CT genotype were 27 kg heavier than animals with the TT genotype (P *=* 0.0300).

**Table 2:** Least square means (LSM) ± standard error (SE) of individual effects of evaluated SNPs on growth traits in Charolais cattle

Trait	Loci	P-value	n	Genotype	LSM	SE	MC
BW	rs210778604	0.0486	41	CC	47.136	2.883	b
			67	CT	46.131	2.677	b
			29	TT	49.990	2.917	a
WW	g.106,004,449	0.0028	0	CC	-	-	
			96	CT	234.443	11.481	a
			35	TT	213.714	12.314	b
	rs208140993	0.0243	18	TT	246.334	14.342	a
			48	TC	230.394	12.179	b
			72	CC	220.772	11.728	b
	rs385131275	0.0059	71	GG	231.393	11.725	b
			60	GA	219.081	11.758	b
			7	AA	260.889	17.546	a
YW	g.106,004,449	0.0300	0	CC	-	-	
			72	CT	420.272	26.108	a
			21	TT	392.381	27.821	b
	rs208140993	0.0695	16	TT	441.990	28.998	a
			64	TC	412.568	26.611	b
			49	CC	411.772	26.397	b
FS	rs210778604	0.0195	32	CC	119.651	2.810	a
			57	CT	118.557	2.531	b
			23	TT	114.363	2.855	b

**Table 3:** Novel and reported SNPs for association analysis

Gene	Gen location	Allele	Amino acid (AA)	Change of AA class	AA position	SNP ID^a^
RXRA	Exon 1	[C/T]	Pro (P)	Ser (S)	8	rs209839910
	Intron 1	[A/G]	------	------	------	g.105,985,004
	Intron 1	[G/T]	------	------	------	g.105,985,027
	Intron 1	[T/G]	------	------	------	g.105,985,044
	Intron 1	[G/A]	------	------	------	g.105,985,130
	Intron 1	[C/T]	------	------	------	g.105,986,006
	Exon 2	[A/G]	Ser (S)	No change	136	g.105,986,149
	Exon 3	[G/T]	Val (V)	No change	148	g.105,986,715
	Exon 3	[A/C]	Asn (N)	Thr (T)	162	rs137184653
	Exon 4	[C/T]	Pro (P)	Leu (L)	198	g.105,989,022
	Exon 4	[C/T]	Thr (T)	No change	217	g.105,989,080
	Intron 4	[G/A]	------	------	------	g.105,989,114
	Intron 4	[A/G]	------	------	------	g.105,989,179
	Intron 4	[T/C]	------	------	------	g.105,989,219
	Intron 4	[T/C]	------	------	------	g.105,989,236
	Intron 4	[G/A]	------	------	------	g.105,989,283
	Exon 5	[G/A]	Arg (R)	Lys (L)	245	g.105,989,790
	Intron 5	[G/A]	------	------	------	g.105,989,983
	Intron 5	[A/C]	------	------	------	g.105,990,023
	Exon 7	[G/A]	Pro (P)	No change	357	g. 105,990,568
	Exon 8	[T/C]	Arg (R)	No change	370	rs136289117
	Intron 9	[G/A]	------	------	------	g.106,004,142
	Intron 9	[A/C]	------	------	------	g.106,004,147
	Intron 9	[G/A]	------	------	------	g.106,004,180
	Intron 9	[A/G]	------	------	------	g.106,004,184
	Intron 10	[T/C]	------	------	------	g.106,004,449
	Intron 10	[C/A]	------	------	------	g.106,004,518
	Intron 12	[G/A]	------	------	------	g.106,009,252
	Intron 12	[G/A]	------	------	------	g.106,009,293
	Intron 12	[C/T]	------	------	------	g.106,011,088
	Intron 12	[C/G]	------	------	------	g.106,011,096
	Intron 12	[T/G]	------	------	------	g.106,011,126
	Exon 13	[C/T]	Ile (I)	No change	667	g.106,011,238
	Exon 13	[C/T]	Pro (P)	No change	672	g.106,011,253
	Intron 13	[C/T]	------	------	------	g.106,011,448
	Intron 13	[C/T]	------	------	------	g.106,011,466
	Intron 13	[G/A]	------	------	------	g.106,011,539
IGF1R	Exon 1	[T/C]	Ser (S)	No change	4	rs379619394
	Exon 1	[A/G]	Gly (G)	Arg (R)	6	rs385718425
	Exon 1	[C/A]	Leu (L)	Ile (I)	25	rs209595810
	Exon 1	[T/G]	Ile (I)	Met (M)	28	rs380419725
	Exon 1	[T/G]	Ser (S)	Ile (I)	29	rs378266791
	Exon 2	[T/C]	Cys (C)	No change	33	rs134868883
	Exon 7	[T/C]	Asp (D)	No change	491	rs41961336
	Exon 7	[A/C]	Thr (T)	Pro (P)	496	rs135514117
	Exon 7	[C/T]	Ser (S)	Pro (P)	497	rs132825686
	Exon 8	[C/T]	Ala (A)	No change	583	rs385548776
	Exon 10	[C/T]	Asp (D)	No change	675	rs210778604
	Exon 11	[C/T]	Thr (T)	No change	773	rs209736678
	Exon 12	[G/A]	Pro (P)	No change	837	rs41640706
	Exon 13	[C/T]	Ser (S)	No change	881	rs133373507
	Exon 16	[C/T]	Tyr (Y)	No change	987	rs208140993
	Exon 19	[G/A]	Lys (K)	No change	1168	rs380900001
	Exon 21	[G/A]	Ser (S)	No change	1308	rs384753755
TCF15	Exon 1	[C/G]	Asp (D)	Glu (E)	32	rs134079367
	Exon 1	[T/G]	Ser (S)	Ala (A)	37	rs137532487
	Exon 1	[A/C]	Gln (Q)	Pro (P)	73	rs134702498
	Intron 1	[G/A]	------	------	------	g.60,997,442
INS	Exon 1	[G/A]	Ala (A)	Thr (T)	24	rs383254521
	Intron 1	G/A	------	------	------	g.50,036,892
	Intron 1	C/T	------	------	------	g.50,036,987
	Intron 1	A/G	------	------	------	g.50,037,033
	Exon 2	[T/G]	Val (V)	Gly (G)	63	rs135743222
	Exon 2	[C/T]	Pro (P)	Leu (L)	72	rs109490717
	Exon 2	[C/T]	Pro (P)	Leu (L)	80	rs109229312
EGFR	Exon 1	[T/C]	Lys (K)	Arg (R)	29	rs136877925
	Exon 4	[T/C]	Asn (N)	Asp (D)	182	rs135955902
	Exon 6	[T/G]	His (H)	Pro (P)	233	rs137416447
	Exon 7	[A/G]	Asn (N)	No change	280	rs209095847
	Exon 8	[C/T]	Val (V)	Ile (I)	318	rs211658253
	Exon 18	[T/C]	Ser (S)	No change	720	rs210170316
	Exon 21	[T/C]	Ala (A)	No change	839	rs110045273
	Exon 25	[G/A]	Arg (R)	No change	999	rs379217506
	Exon 28	[G/A]	Val (V)	No change	1107	rs385131275
USF1	Intron 2	A/G	------	------	------	g.8,458,558
	Intron 3	G/A	------	------	------	g.8,458,837
	Intron 6	A/G	------	------	------	g.8,459,971
	Exon 8	C/T	Ser (S)	No change	236	g.8,460,354
	Intron 9	C/T	------	------	------	g.8,460,878

^a^Based on GenBank *Bos taurus* genomic sequence: 507554 (*RXRA*), *IGF1R* (281848), *TCF15* (518491), 280829 (*INS*), 407239 (*USF1*), and 407217 (*EGFR*)

For rs208140993 located in the *IGF1R* gene, animals harboring the TT genotype had higher WWs than those with complementary genotypes (P *=* 0.0243). Finally, the rs385131275 marker in the *EGFR* gene was significantly associated with WW. Animals with the AA genotype exhibited WWs that were 40 and 30 kg higher than those of the heterozygous (GA) and homozygous (GG) genotypes, respectively.

## Discussion

The network generated in this research presented significant differences from the interaction networks previously reported for *B. taurus*. Differences were observed in the sources of information, the methods applied to construct the networks and their coverage, and the number of established interactions. For example, in 2011, Lim et al. [7] employed a literature mining tool to predict genes specifically associated with marbling in cattle and derived two networks primarily associated with the characteristic of interest based on the orthologous relationship between *B. taurus* and *Homo sapiens* (interologous method). The first network demonstrates high reliability and consists of 52 genes. Among these genes, 61 interactions were established. The second network is a widespread network composed of 1090 genes and 1517 interactions. After a topological analysis, 20 genes (with a node degree ≥ 25) were selected as candidate genes related to bovine marbling. Five of these genes were associated with bovine marbling when the expression profile of each gene was evaluated.

Similarly, Hulsegge et al. [10] prioritized candidate genes for reproductive characteristics in cattle based on PPIs reported for existing orthologous genes between *B. taurus* and *H. sapiens* in the STRING database. The genes were prioritized using the average of two calculated scores. The first score was based on the expression profiles of each gene. The second score was based on a literature search. An enrichment analysis was performed using the Database for Annotation, Visualization and Integrated Discovery (DAVID), and represented biological processes were observed. In this work, 59, 89, 53, 23 and 71 candidate genes were identified with associations with reproductive traits in the amygdala, dorsal hypothalamus, hippocampus, anterior pituitary and ventral hypothalamus, respectively.

Moreover, the coverage values established in BosNet (16,065 genes and 1,747,160 interactions, equivalent to 73 % coverage) were higher than the values estimated by Lim and Hulsegge (4.9 and 27 %, respectively). Thus, BosNet relies on the concept of functional interaction networks and the integration of a wide variety of heterogeneous biological data (orthology relationships with different organisms, interactions reported in various databases, correlations between expression levels, similarities between nucleotide sequences, and shared functional domains), whereas the above-mentioned networks were based on data extracted from only a few sources of information.

In BosNet*,* each integrated experiment, whether genetic or computational, added evidence for gene associations; thus, a greater number of genes and biological processes could be represented, which improved the coverage and precision of the network [11]. This improvement is evident in the results plotted in the ROC curves, which assess the predictive power of each of the networks derived for *B. taurus*. The networks derived from a single source of information exhibit a low level of predictive power, low coverage and a reduced number of interactions relative to the networks generated through the integration of diverse biological data. The coverage (27 %) obtained by Hulssege et al. [10] is noteworthy because the coverage was greater than that achieved in previously reported networks and exhibited greater predictive power than STRING (AUC 0.51) in this study, which was similar to the performance obtained in the integrated network BosNet (AUC 0.64). These results were expected because the interactions in STRING were generated using an integrative method that is conceptually similar to the methodology applied in the present study [12]. Another important point is that the predictive power (i.e., ROC curve) of the networks reported for *B. taurus* that indicates the ability of each of these networks to correctly identify genes involved in a particular characteristic have not been assessed.

The coverage and number of interactions established in BosNet are similar to the results of functional interaction networks reported for other organisms of major economic and scientific importance, such as *Oryza sativa*, *Arabidopsis thaliana, Saccharomyces cerevisiae, Caenorhabditis elegans, Mus musculus* and *H. sapiens*, whose coverages range from 50 to 95 % of the genes reported for each of the organisms, with the number of established interactions ranging from 100,000 to 1.7 million [11, 13–17].

Currently, the availability of different types of biological data, such as functional annotations for *B. taurus* genes, is limited compared with the information available for more thoroughly studied organisms, such as *H. sapiens* [10].

Recently, systems biology approaches have revealed that genes associated with the same or related phenotypes tend to participate in common functional modules (such as protein complexes and metabolic pathways). Moreover, the analysis of protein interaction networks and the neighborhood of a given protein within the network have been used to functionally characterize proteins (guilt-by-association approach).

The guilt-by-association strategy has been widely applied. For example, Lee et al., in 2008 [13], 2010 [18] and 2011 [16], identified genes directly associated with different phenotypes in *C. elegans*, *A. thaliana* and *O. sativa*, respectively, through an analysis of functional interaction networks.

Due to high genetic variation in the genome, SNPs have become the most useful type of marker for gene mapping and association studies. In bovines, different strategies have been used to discover SNPs and assess SNP associations with ERTs. Lee et al. [19] reported a pipeline to analyze non-synonymous SNPs in *B. taurus* after screening the SNPs, which were reported as coding SNPs (cSNPs). They detected 15,353 candidate cSNPs and established a panel of 41 SNPs to evaluate associations with puberty age, facial eczema resistance and meat yield. Three SNPs were nominally associated with facial eczema resistance (P < 0.01).

Commercial arrays in genome-wide association studies (GWAS) have been widely used to understand the genetic basis of complex traits in *B. taurus*; however, the genetic variation underpinning these traits cannot be exclusively explained by this approach. High-throughput sequencing technology could serve as an alternative, but sequencing large numbers of individual genomes remains prohibitively expensive.

Here, we used BosNet to prioritize novel and reported genetic variation in six candidate genes based on SNPs and performed an association study for growth traits.

Because *IGF1R* is established in the bovine somatotropic axis, the *IGF1R* gene is one of the only BosNet-prioritized candidate genes that was previously associated with bovine growth traits. The *IGF1R* gene is the primary receptor for insulin-like growth factors (*IGF*s), which perform the metabolic signal transduction responsible for cell proliferation, bone growth and protein synthesis in the *GH-IGF* pathway.

The *IGF1R*/*Taq I* polymorphism in one of the introns of this gene, which was identified by Moody et al. [20], has been analyzed in several studies but has not been associated with growth traits. Researchers have concluded that this lack of association is caused by the absence of one of its alleles in *B. taurus*; its low frequency in *B. indicus*; and its location on chromosome 21, which is one of the least favorable chromosomes for finding loci associated with growth and carcass composition [21–23]. Here, we identified novel polymorphic markers in *IGF1R* both in Charolais and Brahman cattle. Of these markers, rs210778604 and rs208140993, located in the *IGF1R* coding regions, were significantly associated with BW/FS and WW, respectively. However, validation of these results with a higher number of animals is required.

The *RXRA* gene produces a protein that belongs to a family of transcription factors and plays an important role in fat storage and movement. In knockout mice, this transcription factor demonstrated resistance to obesity induced by chemicals that can be found in diets. Adipogenesis and lipolysis were also affected [24]. This gene demonstrated high genetic variation in the studied populations. We confirmed at least 20 SNPs. SNP g106,0040,449 demonstrated a significant association with WW and YW in the Charolais population. BW is correlated with calving ease and survival, and WW is a reliable index of adult weight performance and productive efficiency [25]. Therefore, confirmation of the association is important to include this marker as a tool for marker-assisted selection based on these traits.

Finally, *EGFR*, which is located on the cell surface, is a mediator of cellular proliferation and differentiation. The binding of its ligand activates a tyrosine kinase that phosphorylates various substrates, thus activating pathways promoting cell growth and DNA synthesis [26]. Here, we found that animals with the AA genotype for the rs385131275 marker from the *EGFR* gene exhibited WWs that were 40 and 30 kg higher than those of animals with heterozygous (GA) and homozygous (GG) genotypes, respectively.

Insulin is a polypeptide hormone produced and secreted by the beta cells of the islets of Langerhans in the pancreas. Insulin improves the absorption of glucose in cells. Qui et al. [27] proposed insulin gene as a candidate gene for the genetic analysis of complex traits, such as growth rate, body composition and fat deposition, in chickens. They analyzed the associations of four polymorphisms located in non-coding regions with 13 different characteristics of growth and body composition. Their findings indicated that one of the polymorphisms and a combination of haplotypes were significantly associated with BW adjusted to 28 days.

Here, we confirm polymorphisms of novel and previously reported SNPs located in the bovine *INS* gene. However, no association with the analyzed growth traits was observed.

The participation of the remaining candidate genes (i.e., *USF1* and *TCF15*) in bovine growth could be deduced based on the function established for each of the genes (no association results for this trait were identified in this study, and none have been identified in cattle to date). In mice, the *TCF15* gene revealed that this transcription factor is an important regulator of a subset of myogenic cells of the dorsolateral dermomyotome associated with the formation of non-migratory hypaxial muscles (abdominal and intercostal) [28]. Moreover, *USF1* is a transcription factor that has been suggested to act as a negative regulator of cell proliferation because it competes for DNA binding sites with transcription factors, such as Myc, which is involved in transformation, cellular proliferation and apoptosis [29, 30].

From a panel of 79 SNPs, we determined that markers rs210778604 and rs208140993 (located in the *IGF1R* coding regions) were associated with BW/FS and WW, respectively (Table 2). In addition, markers rs385131275 and g.106,004,449 (located on the *EGFR* and *RXRA* genes, respectively) were significantly associated with WW and YW in Charolais cattle.

The number of nominally significant associations and the strength of these associations with growth traits were compared to the results obtained from studies that applied the GWAS approach to identify markers associated with growth traits [31]. Thus, BosNet can be used as a prioritization tool to direct the search for novel SNPs that are potentially associated with ERTs.

Updating BosNet is a dynamic process that adds new genes and increases the robustness of each represented biological process. Thus, novel interactions appear that may change the prioritization weighting of each interaction net. Because of this effect, BosNet users must consider that after an update, genes prioritized with a previous version of BosNet may no longer receive prioritization, even if they are still part of the interaction. Here, we use data from the 2012 version of BosNet, as it was at that time that we initially prioritized all the candidate genes that were genotyped and associated with growth traits. According to our records, the prioritization weightings for these genes did not change significantly from those obtained using the BosNet version updated in December 2014; however, in the current version of BosNet (March 2015), none of the previously prioritized genes reached the confidence threshold. We are currently working to improve the network topology analysis. Meanwhile, BosNet users must consider the uniformity of the selected candidate genes and favor those genes that increase the number of strong interactions.

## Conclusions

By integrating heterogeneous biological data, a functional interaction network, BosNet, was constructed for *B. taurus*; BosNet provides 73 % coverage of the estimated genes in the bovine genome.

The transfer of functional Gene Ontology BP annotations to *B. taurus* genes from orthologous genes in more extensively studied organisms increased the coverage and precision of the integrated network compared with the exclusive use of Gene Ontology annotations reported for *B. taurus*.

*INS, TCF15, IGF1R, RXRA, EGFR* and *USF1* were identified as candidate genes associated with bovine growth traits through a search guided by BosNet. Re-sequencing of the coding regions of the candidate genes *INS, USF1, TCF15* and *RXRA* identified three, five, one and 34 new SNPs, respectively, as candidates associated with phenotypic variation of bovine growth traits. From these novel SNPs, associations with growth traits were identified in Brahman and Charolais cattle.

## Methods

### Construction of a functional network for B. taurus

**Fig. 1:**
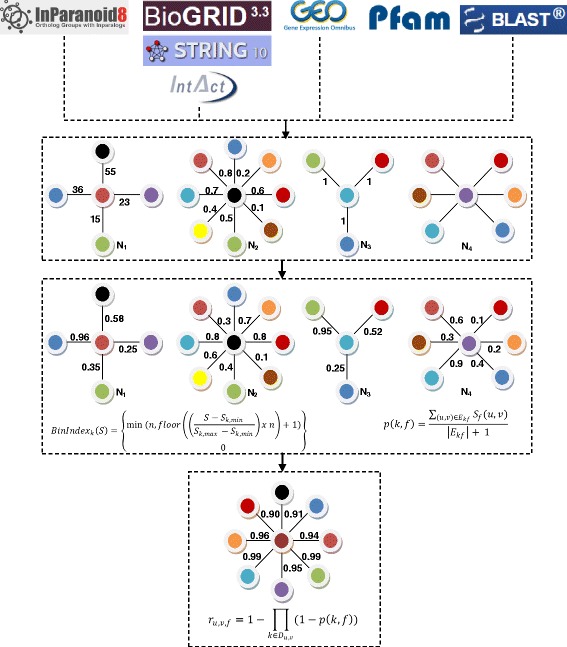
BosNet construction. Information compiled from the different databases was modeled as an undirected graph (N1, N2, N3, N4). Each of the nodes and vertices represents an interaction between a protein pair. The score associated with the graph interaction from each database is represented by a different specific source (i.e., expression level, sequence homology, or conserved domains). Because of differences in the measurement scales, standardization was required. New scores were assigned according to the reported functional annotations (Gene Ontology) between interacting proteins. Finally, the different graphs were integrated to create an integrated functional network of interactions between proteins. The final scores were calculated by assigning greater values to interactions that were represented in more than one database

As shown in Fig. 1, different databases were analyzed, and information related to *B. taurus* was extracted for modeling in an undirected graph *G =* (*V*, *E*), where *V* and *E* are a set of vertices and edges in *G*. Each vertex represents a protein, and each edge (*u, v*) represents an association between proteins.

To provide a better confidence weighting between the interactions, a normalization procedure was used. Given a set of interactions *E* (network) from a *k* data source where the vertices of each edge *E* have at least one functional annotation, *E* was subdivided into subsets using the following approach:

- The *E* interactions were analyzed to find the maximum and minimum scores, *S*_*k,max*_ and *S*_*k,min*_, respectively.
- The *E* interactions were ordered in n subsets *b*_*1*_*.....bn*, with equal intervals between *S*_*k,max*_ and *S*_*k,min*_.
- Each *b*_*i*_ subset was used as a different subtype for which confidence was assessed individually using equation (1).

Given an observation *O*_*e,k,S*_ and interaction data source with an S value *k*, the subset or subtype was determined as follows:

1 $$ BinInde{x}_k(S)=\left\{\begin{array}{c}\hfill \min \left(n, floor\left(\left(\frac{S-{S}_{k, min}}{S_{k, max}-{S}_{k, min}}\right)x\ n\right)+1\right)\hfill \\ {}\hfill \kern3em 0\hfill \end{array}\right\} $$

*Si S* ≥ *S*_*k,min*_

*Si S* < *S*_*k,min*_

- S ≥ *S*_k,min_ and S < *S*_*k,min*_ represent the requirements that each evaluated score must meet. The score may be greater than, less than or equal to the minimum score value in the net.
- If *S ≥ S*_*k,min*_, the *e* confidence based on observation *O*_*e,k,S*_ is calculated by the confidence of each subtype defined by *BinderIndex*_*k*_(*S*).
- Given that *S*_*k,min*_ is determined by the test data based on interactions in which both vertexes are recorded, it is possible that S may be smaller than *S*_*k,min*_. If *S < S*_*k,min*_, the *e* confidence based on the *O*_*e,k,S*_ observation is considered to be 0 because it is not possible to determine its confidence.
- *The floor represents the n subset in the k database to which each evaluated score belongs.*

All of the interactions’ confidence values were re-calculated by subset and database using BP domain of Gene Ontology (http://www.geneontology.org/) (The Gene Ontology Consortium, 2000) as a common criterion. Annotations associated with *B. taurus* genes (~34,082) in the BP domain were downloaded in November 2012.

The interaction confidence was calculated using equation 2:

2 $$ p\left(k,f\right)\kern0.5em =\kern0.5em \frac{{\displaystyle {\sum}_{\left(u,v\right)\in {E}_{kf}}}{S}_f\left(u,v\right)}{\left|{E}_{kf}\right| + 1} $$

*E*_*kf*_ is the interaction subset from *k* database, where each interaction has one or both vertexes annotated with *f* function and both vertexes have at least one functional annotation.

*S*_*f*_(*u*, *v*) = 1 if *u* and *v* share a function or 0 otherwise.

Multiple graphs constructed from the different databases were combined to obtain a unique graph (G') that includes all nodes and their associations. The confidence of each interaction (*u,v*) in G' was calculated using equation 3:

3 $$ {r}_{u,v,f}=1-{\displaystyle {\prod}_{k\in {D}_{u,v}}\left(1-p\left(k,f\right)\right)} $$

*D*_*u,v*_ is the set of databases that have interactions (*u,v*).

Using the algorithm INPARANOID (http://inparanoid.sbc.su.se/) [32], orthologous gene groups were identified between *B. taurus* and other organisms, such as *H. sapiens, M. musculus, C. elegans, A. thaliana, O. sativa* and *S. cerevisiae*. The functional networks for each of these organisms were downloaded from the FunctionalNet server (http://www.functionalnet.org/): HumanNet v.1 [15], MouseNet v.1 [14], WormNet v.2 [13], AraNet v.1 [18], RiceNet v.1 [16] and YeastNet v.2 [33]. From each of these functional networks, a *B. taurus* network was derived using an interologous approach [34], and the value previously associated with each of these interactions served as the score of the association.

Data from four microarray experiments conducted in *B. taurus* were downloaded from Gene Expression Omnibus (GEO) (http://www.ncbi.nlm.nih.gov/geo/info/faq.html) [35]: GSE25005 [36], GSE23837 [37], GSE19055 [38] and GSE35185 [39]. Using GEO2R (http:/www.ncbi.nlm.nih.gov/geo/geo2r), differentially expressed genes were identified with an adjusted p*-*value ≤ 0.05. We combined the above-mentioned DNA microarray experiments to create a single, consistent expression vector for each differentially expressed gene and then measured the Pearson correlation coefficient between these mRNA expression vectors. Thus, a pair of genes was connected with an edge if the Pearson’s correlation coefficient was ≥ 0.7. This value was also used as a confidence score associated with each interaction.

The BioGRID (http://www.thebiogrid.org) [40], STRING (http://string.embl.de/) [12] and IntAct (http://www.ebi.ac.uk/intact/) [41] databases were downloaded in December 2014. These databases list the interactions between proteins derived from different methods; thus, the proteins are already associated in networks. For this reason, only existing interactions between *B. taurus* proteins were extracted.

Information assigned to the proteome functional domains of *B. taurus* was downloaded in December 2014 from the Pfam database (http://pfam.sanger.ac.uk) [42]. An association between two proteins was considered to exist if they shared at least one functional domain. The number of shared domains between each protein was used to represent the score associated with each interaction.

The sequences reported for proteins in the *B. taurus* genome (23,657) were downloaded from the National Center for Biotechnology Information (NCBI) (http://www.ncbi.nlm.nih.gov/). Using the BLAST application (http://blast.ncbi.nlm.nih.gov/Blast.cgi), a database was created to perform BLAST searches with the downloaded sequences. Using blastp, each of the reported *B. taurus* protein sequences was compared with the generated database. To model this information as a network, an association between two proteins was established when their alignment length was ≥ 50 % of the length of the query protein. The percentage of similarity was ≥ 40 %, and the *e-*score was < 0.0001. The negative logarithm of the *e*-score was used for the associated score of each interaction.

The 15 *B. taurus* networks derived using the different methods and databases were integrated via the strategy reported by Chua et al. [43], namely, Integrated Weighted Averaging (IWA). The subset size was 10. To recalculate the associated scores, Gene Ontology (http://www.geneontology.org/) [44] annotations associated with *B. taurus* genes (~32,082) in the BP domain, which was downloaded in November 2012, were used.

Approximately 8243 bovine genes lacked a functional Gene Ontology BP annotation, which directly affected the number of genes that were integrated and the quality of the predictions. To counter this effect, the *B. taurus* genes without annotations were assigned functional Gene Ontology annotations based on orthology. Thus, orthologous groups of genes present in *H. sapiens, M. musculus, C. elegans,* and *S. cerevisiae* were identified, and annotations that were present in each of these organisms were identified and transferred to the genes in question. BosNet was generated by integrating all of the information (Fig. 1).

### Identification and prioritization of candidate genes for growth traits

Genie software (http://cbdm.mdc-berlin.de) [45] was used to perform PubMed based-text mining of genes that were previously associated with bovine growth traits (reference genes).

To identify and prioritize candidate genes for each of the integrated networks, the interactions of the reference genes were extracted, and the degree of association with growth (DAG) was calculated for each of the genes in the following subnet.

$$ DAG={\displaystyle {\sum}_{j\ \in\ ref\  genes}{W}_{ij}}\ .\kern0.5em {\displaystyle {\sum}_{j\ \in\ ref\  genes}{P}_{ij}} $$

where W_ij_ is the linkage weight connecting protein *i* and reference protein *j* and P_ij_ is the number of links connecting protein *i* and reference protein *j* (excluding itself). Thus, the probability that each of these proteins is associated with growth was evaluated based on the protein’s interaction with genes whose biological function had already been associated with this trait.

Using this information, the predictive power of each of the modeled networks for *B. taurus* was evaluated, and the ability of these networks to correctly identify genes associated with growth was measured. This predictive power was characterized using ROC curves. The AUC was used as an indicator of the predictive power. AUC values ≤ 0.5 represent random predictions; AUC values > 0.5 represent predictions ranging from average to good.

For the selection of candidate genes involved in phenotypic variations in growth traits, the new score was used to calculate PPV, which indicates the likelihood of gene association with the growth trait [46]. The selection criterion for candidate genes to be associated with bovine growth was a PPV greater than 0.5 (genes with a greater than 50 % probability).

### Discovery and association of SNPs located in prioritized genes with growth traits

The DNA of two populations was used to conduct the experimental evaluations in this work. All sampling procedures were approved by the Institutional Investigation Ethics Committee (Escuela Superior de Medicina, IPN). The SNP discovery population consisted of nine individuals from varying breeds based on their genetic background and productive purpose (three Holstein, three Brahman and three Charolais). The second group of animals included 237 animals (99 Brahman and 138 Charolais samples). All of the animals were registered, and productive data (weight at birth, weaning and one year of age) were available.

All of the samples were genotyped with 79 SNPs (Table 3) located at the previously prioritized candidate genes using the Sequenom MassARRAY® platform (GeneSeek, Inc., Lincoln, NE, USA). The genotypic and allelic frequencies were estimated using Genepop® 4.0.10 software [47, 48].

Data regarding the growth traits of a 237-animal population of Brahman (n *=* 99) and Charolais (n *=* 138) cattle were used to assess the effect of new and previously identified SNPs by BosNet. Brahman data were fitted using a general linear model procedure that included fixed effects (herd, birth season and sex), random effects (sire and birth year), and the individual effects of genotype in each studied SNP. The adjusted growth traits included BW, WW and YW. Charolais data were only fitted with the fixed effects of sex, season and birth year. For Charolais data, growth traits were also described by analyzing the Frame Size (FS). The least mean squares of the genotypes were estimated for SNPs that demonstrated a significant effect, and a mean comparison was performed using the piecewise differentiable (PDIFF) method. All of the procedures were performed using SAS 9.0 software (SAS Institute Inc., Cary, NC, USA).

## Abbreviations

SNPs: Single nucleotide polymorphisms
ERT: Economically relevant traits
BP: Biological Process
PPV: Positive predictive value
ROC: Receiver operating characteristic curves
AUC: Area under the curve
*RXRA*: Retinoid X receptor alpha
*IGF1R*: Insulin-like growth factor 1 receptor
*TCF15*: Transcription factor 15
*INS*: insulin
*USF1*: Upstream transcription factor 1
*EGFR*: Epidermal growth factor receptor
GWAS: genome-wide association studies
IWA: Integrated Weighted Averaging
DAG: Degree of association with growth
BW: Weight at birth
WW: Weaning weight
YW: One year of age weight
FS: Frame Size
PDIFF: Piecewise differentiable.
